# Soil Nitrogen-Cycling Responses to Conversion of Lowland Forests to Oil Palm and Rubber Plantations in Sumatra, Indonesia

**DOI:** 10.1371/journal.pone.0133325

**Published:** 2015-07-29

**Authors:** Kara Allen, Marife D. Corre, Aiyen Tjoa, Edzo Veldkamp

**Affiliations:** 1 Soil Science of Tropical and Subtropical Ecosystems, Büsgen Institute, Georg-August-Universität Göttingen, Göttingen, Lower Saxony, Germany; 2 Fakultas Pertanian, Agroforestry Centre, Universitas Tadulako, Palu, Sulawesi, Indonesia; Agricultural Research Service, UNITED STATES

## Abstract

Rapid deforestation in Sumatra, Indonesia is presently occurring due to the expansion of palm oil and rubber production, fueled by an increasing global demand. Our study aimed to assess changes in soil-N cycling rates with conversion of forest to oil palm (*Elaeis guineensis*) and rubber (*Hevea brasiliensis*) plantations. In Jambi Province, Sumatra, Indonesia, we selected two soil landscapes – loam and clay Acrisol soils – each with four land-use types: lowland forest and forest with regenerating rubber (hereafter, “jungle rubber”) as reference land uses, and rubber and oil palm as converted land uses. Gross soil-N cycling rates were measured using the ^15^N pool dilution technique with in-situ incubation of soil cores. In the loam Acrisol soil, where fertility was low, microbial biomass, gross N mineralization and NH_4_^+^ immobilization were also low and no significant changes were detected with land-use conversion. The clay Acrisol soil which had higher initial fertility based on the reference land uses (i.e. higher pH, organic C, total N, effective cation exchange capacity (ECEC) and base saturation) (P≤0.05–0.09) had larger microbial biomass and NH_4_^+^ transformation rates (P≤0.05) compared to the loam Acrisol soil. Conversion of forest and jungle rubber to rubber and oil palm in the clay Acrisol soil decreased soil fertility which, in turn, reduced microbial biomass and consequently decreased NH_4_^+^ transformation rates (P≤0.05–0.09). This was further attested by the correlation of gross N mineralization and microbial biomass N with ECEC, organic C, total N (R=0.51–0. 76; P≤0.05) and C:N ratio (R=-0.71 – -0.75, P≤0.05). Our findings suggest that the larger the initial soil fertility and N availability, the larger the reductions upon land-use conversion. Because soil N availability was dependent on microbial biomass, management practices in converted oil palm and rubber plantations should focus on enriching microbial biomass.

## Introduction

Lowland tropical forests of Southeast Asia are considered some of the most diverse and carbon rich forests in the world; however, degradation and subsequent conversion of these forests is occurring at rapid rates. In 2012, Indonesia surpassed Brazil in total forest loss, losing 0.84 million hectares (ha) of forest of which 51% were lowland forests [1]. Sumatra, Indonesia, has been enduring deforestation for decades. Over the past 30 years, Sumatra island alone has lost on average approximately 550,000 ha of forest per year with 85% of these losses occurring in lowland regions [2]. Lowland rainforests are especially vulnerable to degradation and conversion because of easy access and a majority of these forests have been converted to economically viable agricultural systems in order to keep up with the world’s growing population and consumption needs. Historically in Sumatra, lowland forests were converted into agroforestry rubber systems, where rubber trees *(Hevea brasiliensis)* were planted within the natural forest landscape [3]. However, this form of agriculture quickly morphed into complete conversion of entire forests into monoculture plantations, such as rubber and more recently oil palm *(Elaeis guineensis)* [4]. From the period 2000–2013, the area of oil palm and rubber has increased by approximately five million ha and one million ha, respectively, across Indonesia [5]. Monoculture plantations will continue to dominate the landscape in Sumatra, with the Indonesian government goal to double oil palm production in the next ten years [6].

Conversion of tropical forests does not only lead to decreases in biodiversity and drive climate change [7], but may also affect the short- and long-term nutrient status of the converted land-use systems [8]. Tropical lowland forests are considered rich in available nitrogen (N), sustaining high N pools and exhibit high soil-N cycling rates [9]. Systems with large pools of available N are vulnerable to large N losses [10]. Forest converted to corn in Sulawesi, Indonesia, exhibits an initial increase in gross N mineralization rates upon forest conversion and is paralleled by increases in soil NO fluxes, N_2_O emissions and N leaching [10]. Over time, these continuously cultivated systems can experience decreases in available N, base cations and overall soil fertility [10–13]. Systems that are N fertilized or combine tree cash crops with N-fixing tree species do not experience a similar decline in soil N availability [11,13], but can experience losses in N via leaching and/or trace gas emissions [10,12].

The internal soil-N cycle consists of processes that produce and retain mineral N in soil. Through quantifying gross rates of soil-N cycling, we can measure separately and compare mineral N production with retention processes, allowing us to understand better the changes in a system’s soil N status. Mineral N production processes, such as gross N mineralization and gross nitrification, indicate soil N availability for both plants and microbial use [14]. Nitrogen immobilization contributes to the retention of mineral N in soil through N assimilation and turnover of soil microbial biomass, and thus minimizing losses [15]. Dissimilatory nitrate reduction to ammonium (DNRA) is also an important retention process in humid tropical forests [16]. DNRA transforms nitrate (NO_3_^-^) to the less mobile ammonium (NH_4_^+^), and the rate of microbial transformation of NH_4_^+^ is larger than the rates of microbial NO_3_^-^ transformation in many tropical forests [17,18]. The most common factors affecting gross soil-N cycling in tropical forests are substrate quality and quantity [13,19], size of microbial biomass pool, availability of soil carbon [20,21], and soil moisture content [22]. These factors, in turn, are influenced by altitude [23], soil age or degree of soil development [24], rainfall, temperature, elevation, presence or absence of organic layer [18,19,25] and soil texture [21,26].

Our study area was located in lowland forest landscapes in Sumatra, Indonesia with highly weathered Acrisol soils and similar climatic conditions. In such landscapes, the most important factor affecting soil N availability is soil texture. Clay soils are known to have higher nutrient ion availability, higher water holding capacity, and higher soil-N cycling rates compared to sandy soils [21,26]. Soils that are well drained (sands and loams) have lower rates of soil-N cycling and lower microbial biomass [21,26]. Therefore even with the added pressure of land-use conversion, soil-N cycling rates and losses should remain low, while the opposite would be expected from the more nutrient rich clay soils. For example, in Brazilian Amazon lowland forests on highly weathered Ferrasol soils, clay soils have higher cation exchange capacity, water holding capacity, microbial biomass and higher soil-N cycling rates or soil N availability than coarse-textured soils [21,26]. These are, in turn, a reflection of the higher soil fertility, plant productivity and decomposition rates in clay Ferrasol soils [26].

On the other hand, because lowland forests are vulnerable to conversion for agricultural use, land-use change and its associated management practices (e.g. fertilization and liming) are additional important factors that can influence soil N availability in converted landscapes. Fertilization (as source of N, phosphorus (P) and potassium (K)) and liming (as source of calcium (Ca) and magnesium (Mg)) may augment the continuous decline of these nutrients with age of converted land uses [27,28]. Studies of gross soil-N cycling processes in lowland Southeast Asian forests are few, while even fewer focus on land-use change effects on gross soil-N cycling processes. Only one study to our knowledge, investigates how land-use conversion affects gross soil-N cycling processes in montane forest soils in Sulawesi, Indonesia. The results of this study illustrate that cacao agroforestry systems exhibit comparable gross NH_4_^+^ transformation rates to the reference forest, which is attributed to N-fixing tree species in such systems providing additional N, while the oldest unfertilized corn sites exhibit the lowest gross NH_4_^+^ transformation rates [13]. Age of converted land use also affects soil N availability with higher NH_4_^+^ transformation rates in younger compared to older unfertilized corn sites [10,13]. Soil-N cycling responses to the combination of land-use change and soil texture have not yet been explored in Southeast Asian lowland forest landscapes.

The aim of our study was to assess changes in soil mineral-N production (gross N mineralization and gross nitrification rates), as indices of soil N availability, and mineral N retention processes (microbial N immobilization and DNRA) with land-use change. Gross soil-N cycling processes were measured in lowland forest and secondary forest with regenerating rubber (hereafter, “jungle rubber”) as reference land uses, and the converted land uses of monoculture rubber and oil palm plantations, all located in two texturally different lowland Acrisol soils in Sumatra, Indonesia. Our study is the first to our knowledge that investigates gross soil-N cycling processes with land-use change in Southeast Asian lowland tropics, while also taking into account soil textural effects. Our investigation also explores the response of soil-N cycling to agricultural management intensity—by comparing systems with no fertilizer or liming input (i.e. jungle rubber and rubber plantations) to those with higher fertilizer and liming inputs, i.e. the controversial biofuel crop, oil palm.

We hypothesized that 1) gross soil-N cycling rates in the reference land uses would be higher in the clay than the loam Acrisol soils, and 2) gross soil-N cycling rates would be higher in the reference land uses (forest and jungle rubber) compared to the unfertilized converted land use (rubber plantation) and would be intermediate in the fertilized converted land use (oil palm plantation). Here, we provide much-needed background information on gross soil-N cycling rates in the dwindling Southeast Asian lowland forests, and how their soil-N production and retention processes are influenced by land-use conversion.

## Materials and Methods

### Study Sites

**Fig 1:**
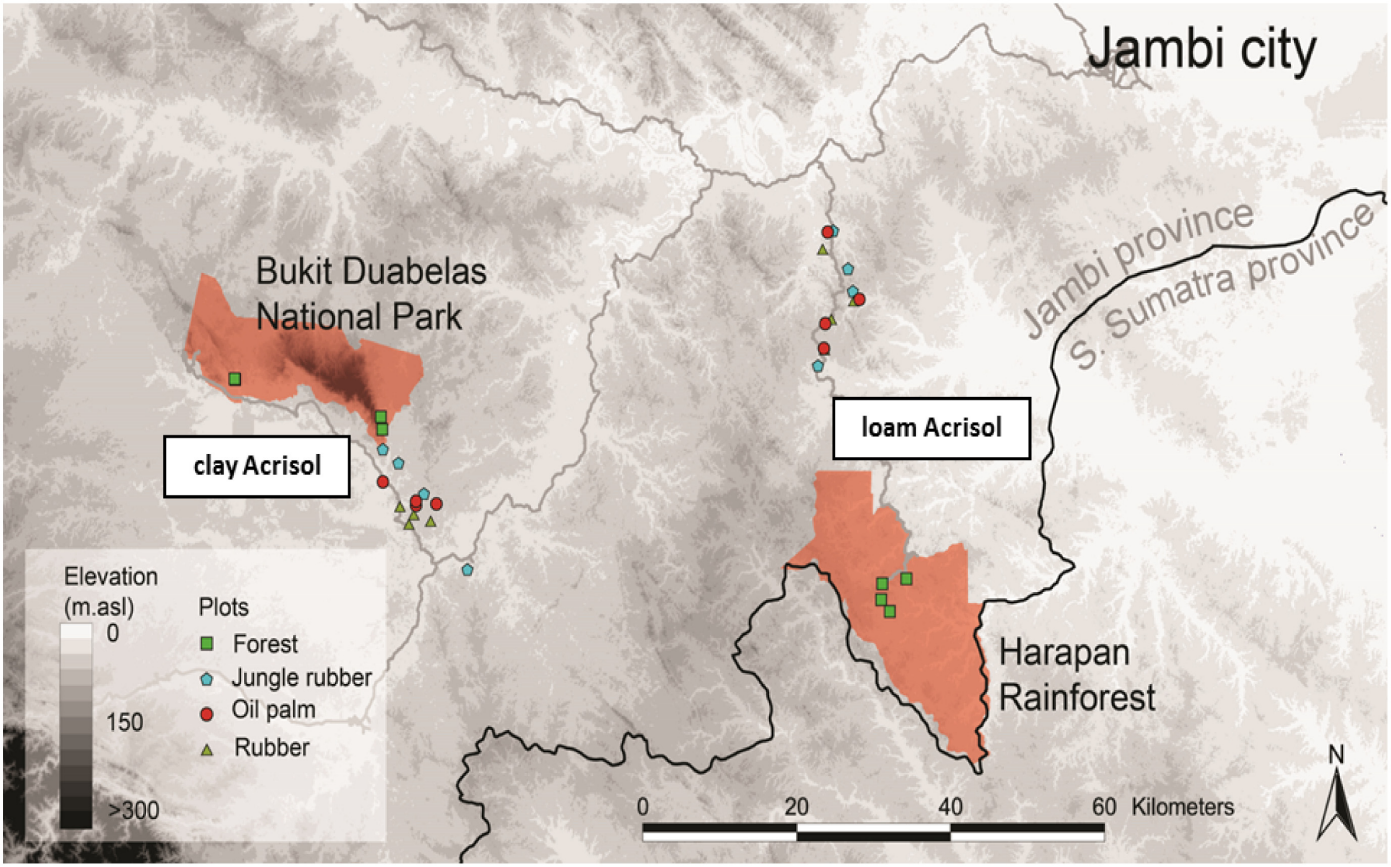
Map of study area located in Jambi, Sumatra, Indonesia. Each of the four land-use types were represented with four replicate plots and plots were clustered in two different landscapes classified based on dominant soil texture and soil type: clay Acrisol soil (located in Bukit Duabelas region with forest sites in the National Park (area shaded in orange)) and loam Acrisol soil (located in Harapan region with forest sites in the PT REKI Harapan protected area (area shaded in orange)). Map created by Oliver van Straaten [43].

The study was carried out in Jambi Province, central Sumatra, Indonesia. Two landscapes, characterized by their dominant soil texture and type, were selected in the region. The loam Acrisol soil (1° 55’ 40” S, 103° 15’ 33” E and elevation of 70 ± 4 m above sea level, asl) was located approximately 60 km south of Jambi city and the clay Acrisol soil (2° 0’ 57” S, 102° 45’ 12” E and elevation of 75 ± 4 m asl) was located approximately 110 km west of Jambi city (Fig 1). Acrisol soils cover 49.9% of the land area in Sumatra and 34.2% in Indonesia [29]. The mean annual temperature is 26.7 ± 1.0°C and mean annual precipitation is 2235 ± 385 mm [1991–2011; climate station at the Jambi Sultan Thaha airport of the Meteorological, Climatological and Geophysical Agency]. Total dissolved N deposition through rainfall ranged from 12.9 ± 0.1 to 16.4 ± 2.6 kg N ha^-1^ yr^-1^ measured in 2013 [Kurniawan et al. unpublished data].

### Experimental and Sampling Design

In each soil landscape, four land-use systems were examined: mixed Dipterocarp [30] lowland forest and forest with regenerating rubber trees or jungle rubber, both as reference land uses, and smallholder monoculture plantations of rubber and oil palm. We consider the forest and jungle rubber as reference, for the baseline conditions that we compared to the converted smallholder plantations, for these reasons: 1) the rubber and oil palm plantations were established after logging, clearing and burning (see *Management Practices in Smallholder Rubber and Oil Palm Plantations* below) of either forests or jungle rubber [Euler et al. unpublished data], and 2) the jungle rubber sites were closer to the monoculture plantations than the forest sites, most of which were located ≥ 10 km from the plantation sites (Fig 1). Trees in the monoculture plantations ranged from 7–17 years old, and tree species diversity, tree density, tree height and basal area [30] were greater in the reference land uses (forest and jungle rubber) than in the converted land uses (rubber and oil palm plantations) (S1 Table).

The space-for-time substitution approach, as used by Corre et al. [15], was employed to determine the effects of land-use change on soil biochemical characteristics and soil-N cycling rates. An implicit assumption of this approach is that the initial soil characteristics were comparable prior to conversion. To test this assumption, we compared land-use independent soil characteristics (i.e. soil texture at deeper depths, ≥ 0.5 m) among land uses within each landscape. Since we did not detect significant differences in soil texture between the reference land uses and the converted plantations within a soil landscape (S2 Table), we have assumed that the soil conditions were previously similar and that observed soil biochemical and soil-N cycling changes can be attributed to changes in land use.

**Fig 2:**
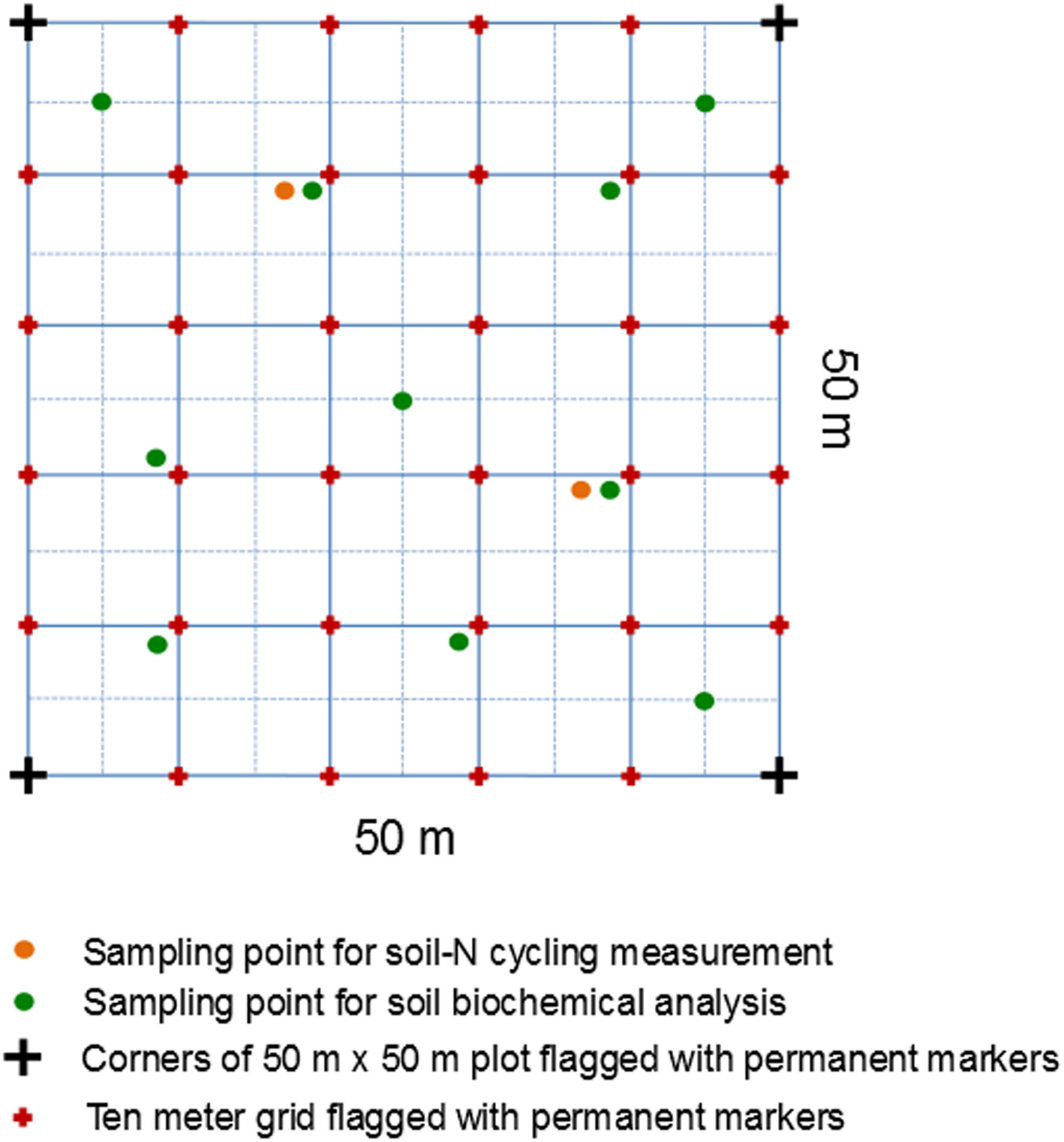
Sampling design in each of the four replicate plots (50 m x 50 m each) of the four land uses in the two soil landscapes (totaling 32 plots). Each plot had a 10 m x 10 m grid. Ten sampling points were selected for soil sampling for biochemical analysis (green dots) and two sampling points were selected for measuring gross soil-N cycling rates (orange dots).

For each land use in each landscape, four replicate plots were selected; each replicate plot was 50 m x 50 m with a minimum distance of 200 m between plots (Fig 1). A 10 m x 10 m grid was established across each plot, and we randomly selected ten grid points as subplots that were at least 5 m distance from the plot’s border for soil sampling (Fig 2). Soil samples were taken within an area of 0.4 m x 0.4 m at each grid point, and were used to measure the general soil biochemical characteristics (see below). Soil characteristics for each replicate plot were represented by the average of the ten individual subplots. Soil sampling was conducted between June 2013 and December 2013. The soil had no organic layer but only a thin litter layer, and this was removed in order to sample predominantly mineral soil.

Soil samples were taken at various depth intervals down to 2 m, and we report here the values from the top depth interval (0–0.1 m), except for clay percent, which we report for the top 0.5 m (Table 1) and depths ≥ 0.5 m (S2 Table). Soil samples were air dried and sieved (2 mm) at the University of Jambi, Indonesia and sent to the Soil Science of Tropical and Subtropical Ecosystems (SSTSE) laboratory at Georg-August University Göttingen, Germany for analysis.

**Table 1:** Soil characteristics (means ± SE, n = 4) in the top 0.1 m depth for different land-use types within each soil landscape in Jambi, Sumatra, Indonesia.

	Land-use types			
	Reference land uses		Converted land uses	
Characteristics	Lowland forest	Jungle rubber	Rubber plantation	Oil palm plantation
	loam Acrisol soil			
Clay (%)^3^	26.0 ± 2.6	30.6 ± 4.6	37.3 ± 10.3	33.4 ± 2.2
Bulk density (g cm^-3^)	1.0 ± 0.0 ab^1^	0.9 ± 0.0 b	1.1 ± 0.1 a	1.2 ± 0.1 a
pH (1:4 H_2_O)	4.3 ± 0.0 b ^†^	4.3 ± 0.0 B^2^ b ^†^	4.5 ± 0.1 ab ^†^	4.5 ± 0.1 a ^†^
Soil organic C (kg C m^-2^)^4^	2.6 ± 0.2	2.7 ± 0.3 B	2.0 ± 0.3	1.8 ± 0.2
Total N (g N m^-2^)^4^	182.9 ± 10.8	186.1 ± 11.0 B	172.6 ± 23.8	145.0 ± 13.5
C:N ratio	14.3 ± 0.2 a	13.7 ± 0.8 a	11.7 ± 0.7 b	12.5 ± 0.5 ab
Effective cation exchange capacity (mmol_c_ kg^-1^)	44.8 ± 5.0	40.6 ± 7.6 B	46.0 ± 5.4	39.5 ± 7.9
Base saturation (%)	10.6 ± 0.5 B b^†^	16.0 ± 2.2 ab^†^	21.1 ± 7.5 ab^†^	27.9 ± 5.4 a^†^
δ^15^N (‰)	4.3 ± 0.2 b	4.5 ± 0.1 b	5.0 ± 0.4 ab	5.4 ± 0.3 a
Extractable phosphorus (g P m^-2^)^4^	0.5 ± 0.1 B	0.7 ± 0.1	0.5 ± 0.1	0.8 ± 0.1
Aluminum (g Al m^-2^)^4^	33.1 ± 3.5	29.6 ± 6.6 B	30.7 ± 4.3	23.5 ± 2.7
Calcium (g Ca m^-2^)^4^	5.5 ± 2.0	6.9 ± 0.8 B^†^	14.5 ± 7.1	18.5 ± 7.4
Iron (g Fe m^-2^)^4^	0.8 ± 0.1 B a	0.3 ± 0.0 B bc	0.3 ± 0.1 c	0.5 ± 0.02 ab
Magnesium (g Mg m^-2^)^4^	1.8 ± 0.1	2.0 ± 0.3 B	3.4 ± 1.4	1.7 ± 0.9
Manganese (g Mn m^-2^)^4^	0.3 ± 0.1	0.4 ± 0.2 B	0.8 ± 0.3	0.5 ± 0.2
Potassium (g K m^-2^)^4^	3.3 ± 0.3	2.6 ± 0.2 B	3.4 ± 0.8	2.1 ± 0.8
Sodium (g Na m^-2^)^4^	0.5 ± 0.1 B c	1.5 ± 0.2 B b	1.4 ± 0.1 b	3.9 ± 1.1 a
	clay Acrisol soil			
Clay (%)^3^	31.4 ± 5.4	47.2 ± 12.4	42.4 ± 3.1	59.7 ± 5.2
Bulk density (g cm^-3^)	1.0 ± 0.1	0.8 ± 0.1	0.9 ± 0.1	0.9 ± 0.1
pH (1:4 H_2_O)	4.2 ± 0.0 b	4.5 ± 0.0 A a	4.5 ± 0.1 a	4.4 ± 0.0 a
Soil organic C (kg C m^-2^)^4^	3.3 ± 0.5	4.3 ± 0.4 A	2.8 ± 0.4	3.5 ± 0.2
Total N (g N m^-2^)^4^	263.4 ± 67.1	331.4 ± 34.1 A	198.9 ± 32.5	260.2 ± 22.6
C:N ratio	13.1 ± 1.3	13.0 ± 0.3	14.3 ± 0.6	13.5 ± 0.2
Effective cation exchange capacity (mmol_c_ kg^-1^)	94.3 ± 40.8	124.5 ± 25.5 A	71.3 ± 22.3	78.1 ± 8.4
Base saturation (%)	22.9 ± 5.6 A	23.2 ± 5.8	20.1 ± 2.6	37.5 ± 7.1
δ^15^N (‰)	4.5 ± 0.0	4.0 ± 0.3	4.6 ± 0.4	5.2 ± 0.4
Extractable phosphorus (g P m^-2^)^4^	1.4 ± 0.1 A ab	0.8 ± 0.1 bc	0.4 ± 0.0 c	4.7 ± 1.5 a
Aluminum (g Al m^-2^)^4^	50.9 ± 22.7	76.6 ± 15.6 A	47.2 ± 17.6	34.4 ± 2.0
Calcium (g Ca m^-2^)^4^	32.3± 21.2	33.3 ± 10.9 A^†^	14.7 ± 2.8	59.1 ± 19.5
Iron (g Fe m^-2^)^4^	3.7 ± 1.1 A a	3.0 ± 0.4 A a	2.3 ± 0.6 a	0.7 ± 0.3 b
Magnesium (g Mg m^-2^)^4^	7.3 ± 3.9	12.0 ± 4.1 A	4.0 ± 0.9	3.5 ± 0.8
Manganese (g Mn m^-2^)^4^	4.5 ± 3.1	2.5 ± 0.7 A	1.5 ± 0.4	3.4 ± 1.3
Potassium (g K m^-2^)^4^	9.4 ± 3.9	9.6 ± 2.6 A	4.2 ± 1.1	4.8 ± 0.9
Sodium (g Na m^-2^)^4^	3.6 ± 0.8 A	4.2 ± 0.2 A	3.7 ± 1.3	1.9 ± 1.3

^1^Within row means followed by different lower case letters indicate significant differences between land-use types within a soil landscape (LME model with Fisher’s LSD test at P ≤ 0.05 and marginally significant at ^†^P ≤ 0.09).

^2^Within column means followed by different upper case letters indicate significant differences between soil landscapes within a reference land use (LME model with Fisher’s LSD test at P ≤ 0.05 and marginally significant at ^†^P ≤ 0.09).

^3^Depth-weighted average for intervals of 0–0.1 m, 0.1–0.3 m and 0.3–0.5 m with n = 3 replicate plots per land use.

^4^Element stocks expressed in g m^-2^ were calculated as: concentrations (g kg^-1^) * average bulk density of the reference land uses in each soil landscape (g cm^-3^) * depth (cm) * 10000 cm^2^ m^-2^ ÷ 1000 g kg^-1^. The average bulk density of the reference land uses is normally used in order to compare the same soil mass and avoid the interference of bulk density changes that often result from land-use changes due to management practices that compact or loosen the soil [42].

For soil-N cycling measurements, we randomly selected two subplots per plot that were at least 10 m from the plot’s border (Fig 2). Soil-N cycling rates for each plot were the average of the two individual subplots. Soil-N cycling measurements were conducted once in all land uses and were completed between January 2013 to May 2013 during the rainy season (see *Gross Rates of Soil-N Cycling* for more details).

### Management Practices in Smallholder Rubber and Oil Palm Plantations

According to interviews with smallholders, conducted by Euler et al. [unpublished data], the rubber and oil palm plantations in the clay Acrisol soil were planted after clearing and burning the previous forest or logged forest. In the loam Acrisol soil, oil palm plantations were established after clearing and burning the previous jungle rubber whereas the rubber plantations were established from previously logged forest. Based on our interviews, only the oil palm plantations were fertilized during our study year, 2013, while the rubber plantations were not. Oil palm plantations in the clay Acrisol soil were fertilized once in the rainy season (October to March), and in the loam Acrisol soil, these were fertilized once in the rainy season and once in the dry season (April to September). The most commonly used fertilizers were NPK complete fertilizer (i.e. Phonska, Mahkota), potassium chloride (KCl) and urea (CO(NH_2_)_2_). Fertilizer additions to the oil palm plantations ranged from 300 kg NPK-fertilizer ha^-1^ year^-1^ (for those plantations that were fertilized once) to 550 kg NPK-fertilizer ha^-1^ year^-1^ (for those plantations that were fertilized twice). In terms of unit nutrient element added, these rates were equivalent to 48–88 kg N ha^-1^ year^-1^, 21–38 kg P ha^-1^ year^-1^ and 40–73 kg K ha^-1^ year^-1^. Additionally, three of the smallholders applied 157 kg K-KCl ha^-1^ year^-1^ and 143 kg Cl-KCl ha^-1^ year^-1^ and two of the smallholders applied 138 kg urea-N ha^-1^ year^-1^. One of the smallholders also applied lime in 2013 at an average rate of 200 kg dolomite ha^-1^ year^-1^. Both manual and chemical weeding took place throughout the year at the rubber and oil palm plantations. The most commonly used herbicides were Gramoxone and Roundup; these were applied at an average rate of 2 to 5 L herbicide ha^-1^ year^-1^ [Euler et al. unpublished data].

### Soil Characteristics

The core method was used to measure soil bulk density for the top 0.5 m of soil depth [31]. Soil pH (H_2_O) was analyzed in a 1:4 soil-to-water ratio. Soil organic C and total N concentrations were analyzed from air-dried, sieved (2 mm) and ground samples using a CN analyzer (Vario EL Cube, Elementar Analysis Systems GmbH, Hanau, Germany). Air-dried and sieved soils were used to determine effective cation exchange capacity (ECEC) by percolating with unbuffered 1 mol L^-1^ NH_4_Cl and cations (Ca, Mg, K, Na, Al, Fe, and Mn) were measured in percolates using an inductively coupled plasma-atomic emission spectrometer (iCAP 6300 Duo VIEW ICP Spectrometer, Thermo Fischer Scientific GmbH, Dreieich, Germany). Base saturation was calculated as percent exchangeable base cations of the ECEC. Extractable P was determined using the Bray 2 method, which is used for acidic tropical soils [32]. For ^15^N natural abundance signatures (δ^15^N), the ten subsamples from each replicate plot were composited, ground and analyzed using isotope ratio mass spectrometry (IRMS; Delta Plus, Finnigan MAT, Bremen, Germany). Soil δ^15^N has been linked to directly reflect soil N availability in that the larger the δ^15^N, the larger the gross rates of mineral N production [18,19].

### Gross Rates of Soil-N Cycling

The ^15^N pool dilution technique on intact soil cores was used to determine gross rates of soil-N cycling processes in the top 5 cm depth [14]. Within each replicate plot, we selected two grid points as subplots that were 20 m apart (Fig 2), and in each subplot we took five intact soil cores (8 cm diameter and 5 cm length) near to each other. Gross soil-N cycling rates (i.e. gross N mineralization, gross nitrification, N immobilization and DNRA) were measured using four of the five soil cores, and background NH_4_^+^ and NO_3_^-^ levels and microbial biomass C and N were measured using one of the five soil cores, described in detail below.

In the field, two soil cores in each subplot were injected with (^15^NH_4_)_2_SO_4_ solution for measurement of gross N mineralization and NH_4_^+^ immobilization and the other two soil cores were injected with K^15^NO_3_ solution for gross nitrification, NO_3_^-^ immobilization and DNRA measurements. Using a side port needle, each intact soil core was injected with 5 mL of ^15^N solution containing 26 ug N- K^15^NO_3_ mL^-1^ and 29 ug N-(^15^NH_4_)_2_SO_4_ mL^-1^ both with 99% ^15^N enrichment. The rates of ^15^N injection for both solutions ranged on average 1.4 ± 0.1 to 2.3 ± 0.1 ug ^15^N g^-1^ across land uses and soil landscapes. One soil core of each ^15^N-injected pair (one with ^15^NH_4_^+^ and one with ^15^NO_3_^-^) was extracted with 0.5 mol L^-1^ K_2_SO_4_ approximately ten minutes after ^15^N injection (T_0_ soil cores). Soil from the core was extruded, mixed well and large roots, stones and woody debris were removed. A subsample was put into a prepared bottle containing 150 mL 0.5 mol L^-1^ K_2_SO_4_ (with approximately 1:3 ratio of fresh soil to K_2_SO_4_ solution). The remaining pair of ^15^NH_4_^+^- and ^15^NO_3_^-^-injected soil cores were placed in a plastic bag and put back into the soil to incubate in-situ for one day (T_1_ soil cores). The plastic bags were closed loosely to allow for air exchange, but prevent rain from entering and potentially leaching ^15^N. T_1_ soil cores were extracted with 0.5 mol L^-1^ K_2_SO_4_ in the same way as the T_0_ soil cores. Part of the soil in each of the T_1_ cores was also fumigated with chloroform (CHCl_3_) upon arrival in the laboratory (within maximally two hours from the field) for six days and then extracted with 0.5 mol L^-1^ K_2_SO_4_. These fumigated T_1_ cores were used for the determination of NH_4_^+^ and NO_3_^-^ immobilization. From each soil core, the remaining soil was oven-dried at 105°C for at least one day to measure gravimetric moisture content, which was then used to calculate the dry mass of the extracted soil.

Upon arrival in the laboratory, soil extraction was continued by shaking the K_2_SO_4_ bottles with soil in a mechanical shaker for one hour. The extracts were filtered through pre-washed (with 0.5 mol L^-1^ K_2_SO_4_) filter papers (4 um nominal pore size), and were frozen immediately. The frozen extracts were transported by air to Germany and remained frozen until further analysis at the SSTSE laboratory.

NH_4_^+^ and NO_3_^-^ concentrations in the extracts were determined by continuous flow injection colorimetry (SEAL Analytical AA3, SEAL Analytical GmbH, Norderstadt, Germany), using a salicylate and dicloroisocyanuric acid reaction for NH_4_^+^ determination (Autoanalyzer Method G-102-93) and the cadmium reduction method with NH_4_Cl buffer for NO_3_^-^ analysis (Autoanalyzer Method G-254-02). ^15^N signatures in the NH_4_^+^, NO_3_^-^ and extractable organic N pools in the extracts were determined by the diffusion method (for organic N, after its conversion to NO_3_^-^ by persulfate digestion), following the same procedures as those outlined by Corre et al. [13,19], and ^15^N was determined using IRMS. Calculations of gross rates of soil-N cycling processes followed the calculation procedures given in detail by Davidson et al. [14] and Silver et al. [16].

From the fifth intact soil core taken at each subplot, initial levels of NH_4_^+^, NO_3_^-^, and soil microbial biomass C and N were determined. For NH_4_^+^ and NO_3_^-^ levels, similar soil sample processing, in-situ K_2_SO_4_ extraction and laboratory analysis were used as above. For microbial biomass, the CHCl_3_ fumigation-extraction method was followed [33,34]. A subsample of soil from a core was extracted immediately with 0.5 mol L^-1^ K_2_SO_4_ (unfumigated) and another subsample was fumigated with CHCl_3_ for six days and then extracted (fumigated) as described above. Organic C in the extracts was analyzed by UV-enhanced persulfate oxidation using a Total Organic Carbon Analyzer (TOC-Vwp, Shimadzu Europa GmbH, Duisburg, Germany) with an infrared detector. Organic N in the extracts was determined by ultraviolet-persulfate digestion followed by hydrazine sulfate reduction using continuous flow injection colorimetry (Method G-157-96; SEAL Analytical AA3, SEAL Analytical GmbH, Norderstedt, Germany). Microbial biomass C and N were calculated as the difference in extractable organic C and N between the fumigated and unfumigated soils divided by k_C_ = 0.45 and k_N_ = 0.68 for a six-day fumigation period [33].

### Statistical Analysis

Statistical analysis was conducted on the means of the ten (soil biochemical characteristics) or two (soil-N cycling) subplots representing each replicate plot. Parameters that exhibited non-normal distribution or heterogeneous variance (tested using Shapiro-Wilk and Levene’s tests) were log transformed. First, we compared between the soil landscapes using the reference land uses to test our first hypothesis, and then among land uses for each soil landscape to test our second hypothesis. Linear mixed effects (LME) models were used with either landscape (i.e. comparing landscapes for each reference land use) or land use (i.e. comparing land uses for each landscape) as the fixed effect and replicate plots as the random effect. For assessing differences in soil-N cycling processes, the LME models included gravimetric moisture content as a covariate, because the measurements of these processes spanned four months (January–May 2013) across all 32 plots during which moisture content slightly varied between landscapes. Using soil moisture content, measured from the same soil cores as the soil-N cycling processes, as a covariate accounts for any masking effect of variation in soil moisture content across the measurement period on the differences among land uses. Fisher’s least significant difference (LSD) test was used to assess significant differences among land uses. Differences were considered statistically significant at P ≤ 0.05. For a few specified parameters, we also considered marginal significance at P ≤ 0.09, because our experimental design encompassed the inherent spatial variability in our study area. To assess the relationships among soil-N cycling processes, we used Spearman’s rank correlation tests among gross rates of soil-N cycling (i.e. gross N mineralization, gross nitrification, N immobilization and DNRA) and microbial (i.e. microbial biomass C and N) parameters within a land use across landscapes. The same correlation test was conducted between gross rates of N mineralization as an index of soil N availability and soil biochemical characteristics across land uses for each landscape to assess how changes in soil biochemical characteristics due to land-use conversion relate to changes in soil N availability. We used R 3.0.3 for all statistical analyses [35].

### Field-work Permission

This study was a part of a DFG (German Research Foundation) funded project: Ecological and socio-economic functions of tropical lowland rainforest transformation systems (EEFForTs). EEFForTs is an interdisciplinary research project that investigates the effects of land-use change on environmental processes, biodiversity, and human dimensions and is a collaboration between Georg-August University Göttingen and several Indonesian universities. Indonesian management teams were established at the University of Jambi and Agricultural University of Bogor and were responsible for assistance in the procurement of all research and collection permits, as well as permissions from local land owners and managers of the agricultural sites for field sampling. This study was conducted using the research permits 215/SIP/FRP/SM/VI/2012 and 44/EXT/SIP/FRP/SM/V/2013 recommended by the Ministry of Research and Technology of the Republic of Indonesia (RISTEK). As well as, the collection permits 2703/IPH.1/KS.02/XI/2012 and S.13/KKH-2/2013 recommended by the Indonesian Institute of Sciences (LIPI) and issued by the Ministry of Forestry (PHKA). The lowland forest sites were located within protected areas managed by the PHKA and Restoration Ecosystem Indonesia Harapan (PT REKI). Indonesian research collaborators have also been involved in all stages from the conceptualization of the EEFForTs project, field design and sampling, sample exportation for analysis in Germany and finally in interpretation of results. The fieldwork did not involve sampling of endangered or protected species, and was predominantly soil sampling for nutrient element analysis.

## Results

### Reference Land Uses: Forest and Jungle rubber

The range of the average clay contents for the reference land uses in the loam Acrisol soil was lower than in the clay Acrisol soil (Table 1). In the forest sites, we did not detect significant differences between the two landscapes in most of the soil biochemical characteristics because of the large spatial variation among sites (e.g. variance components analysis showed that for the forest 32–77% of the variances of soil organic C, total N, C:N ratio and ECEC were due to the variation among plots and only 0–11% were due to the variation between landscapes). This was primarily a result of the greater distance between the two sets of clay Acrisol forest plots (Fig 1). However, a few clear differences between the two landscapes emerged: forest plots in the clay Acrisol soil had higher base saturation, extractable P, exchangeable Fe and Na and lower Al saturation (61 ± 3%) compared to those in the loam Acrisol soil (P ≤ 0.05; Table 1; Al saturation for the loam Acrisol 80 ± 1%). In the jungle rubber, the differences in soil biochemical characteristics between landscapes were clearly shown. Soil pH, soil organic C stocks, total N stocks, ECEC and stocks of exchangeable base (Ca, Mg, K and Na) and acid (Al, Fe and Mn) cations were higher in the clay than loam Acrisol soils (P ≤ 0.05 to 0.09; Table 1). This was due to the lower spatial variation among jungle rubber sites, e.g. variance components analysis showed that only 8–33% of the variances of soil organic C, total N, C:N ratio and ECEC were due to the variation among plots.

The forest sites in the clay Acrisol soil had higher rates of NH_4_^+^ transformation processes (i.e. gross N mineralization and NH_4_^+^ immobilization) and microbial biomass C and N than those in the loam Acrisol soil (P ≤ 0.05; Table 2). There were no significant differences in rates of NO_3_^-^ transformation processes detected between the two landscapes for these reference land uses (P ≥ 0.10); however, the rates of NH_4_^+^ transformation processes were much higher in all land-use types in both landscapes compared to NO_3_^-^ transformation processes (Table 2). Distinguishable attributes in the soil-N cycling between the reference land uses were that the NO_3_^-^ pool was higher in the forest than jungle rubber in both landscapes (both P ≤ 0.05) and the microbial C:N ratio was higher in the forest than jungle rubber in the clay Acrisol soil (P ≤ 0.09; Table 2).

**Table 2:** Gross soil-N cycling rates and pools (means ± SE, n = 4) in the top 0.05 m depth for different land-use types within each soil landscape in Jambi, Sumatra, Indonesia.

	Land-use types			
	Reference land uses		Converted land uses	
N cycling rates or pools	Lowland forest	Jungle rubber	Rubber plantation	Oil palm plantation
	loam Acrisol soil			
NH_4_^+^ (mg N kg^-1^)	2.7 ± 0.4 B^†^ ^2^	2.4 ± 0.1	2.7 ± 0.3	3.2 ± 1.8
Gross N mineralization (mg N kg^-1^ day^-1^)	5.4 ± 0.7 B	4.6 ± 0.6	6.2 ± 0.7	4.2 ± 1.1
NH_4_^+^ immobilization (mg N kg^-1^ day^-1^)	2.7 ± 0.5 B	4.0 ± 1.0	4.3 ± 0.2	1.9 ± 0.4
NO_3_^-^ (mg N kg^-1^)	1.1 ± 0.1 B a^1^	0.4 ± 0.3 bc	0.1 ± 0.1 c	1.4 ± 0.9 ab
Gross nitrification (mg N kg^-1^ day^-1^)	1.9 ± 0.4	0.9 ± 0.2	0.9 ± 0.2	1.2 ± 0.5
NO_3_^-^ immobilization (mg N kg^-1^ day^-1^)	0.9 ± 0.3	0.4 ± 0.2	0.7 ± 0.3	0.6 ± 0.2
DNRA^3^ (mg N kg^-1^ day^-1^)	0.2 ± 0.0 ab	0.2 ± 0.0 b	0.5 ± 0.1 a	0.1 ± 0.0 b
Microbial N (mg N kg^-1^)	69.7 ± 4.8 B	86.5 ± 6.4	73.8 ± 10.9	59.3 ± 6.4
Microbial C (mg C kg^-1^)	514.0 ± 48.4 B	577.7 ± 45.1	461.4 ± 58.1	403.1 ± 23.5
Microbial biomass C:N ratio	7.2 ± 0.3	6.7 ± 0.5	6.3 ± 0.4	7.0 ± 0.4
	clay Acrisol soil			
NH_4_^+^ (mg N kg^-1^)	3.6 ± 0.4 A ab	6.2 ± 1.6 a	2.8 ± 0.2 b	4.3 ± 1.0 ab
Gross N mineralization (mg N kg^-1^ day^-1^)	11.5 ± 1.8 A a^†^	10.8 ± 2.1 a^†^	6.0 ± 0.6 b^†^	9.3 ± 2.1 ab^†^
NH_4_^+^ immobilization (mg N kg^-1^ day^-1^)	16.8 ± 5.7 A a	14.8 ± 2.9 a	5.5 ± 1.2 ab	7.3 ± 3.9 b
NO_3_^-^ (mg N kg^-1^)	1.6 ± 0.2 A a	0.2 ± 0.1 bc	0.1 ± 0.0 c	0.7 ± 0.3 ab
Gross nitrification (mg N kg^-1^ day^-1^)	0.9 ± 0.3	1.0 ± 0.2	0.7 ± 0.2	2.0 ± 0.8
NO_3_^-^ immobilization (mg N kg^-1^ day^-1^)	2.0 ± 0.6	3.3 ± 0.8	1.7 ± 0.6	1.7 ± 0.4
DNRA^3^ (mg N kg^-1^ day^-1^)	0.4 ± 0.2	0.9 ± 0.4	0.5 ± 0.1	0.4 ± 0.1
Microbial N (mg N kg^-1^)	134.4 ± 27.6 A ab	152.8 ± 28.0 a	75.4 ± 6.6 c	104.6 ± 23.4 bc
Microbial C (mg C kg^-1^)	1048.1 ± 200.8 A a^†^	922.3 ± 222.5 ab^†^	560.7 ± 60.7 c^†^	616.6 ± 112.0 bc^†^
Microbial biomass C:N ratio	7.9 ± 0.5 a^†^	5.7 ± 0.6 c^†^	7.5 ± 0.6 ab^†^	6.1 ± 0.4 bc^†^

^1^Within row means followed by different lower case letters indicate significant difference between land-use types within a soil landscape (LME model with Fisher’s LSD test at P ≤ 0.05 and marginally significant at ^†^P ≤ 0.09).

^2^Within column means followed by different upper case letters indicate significant difference between soil landscapes within a reference land use (LME model with Fisher’s LSD test at P ≤ 0.05 and marginally significant at ^†^P ≤ 0.09).

^3^Dissimilatory nitrate reduction to ammonium.

Positive correlations between gross N mineralization, NH_4_^+^ immobilization and microbial biomass were also observed in both reference land uses (Table 3). In the jungle rubber sites, gross N mineralization was positively correlated with NH_4_^+^ and NO_3_^-^ retention processes (i.e. immobilization and DNRA; Table 3).

**Table 3:** Spearman’s rank correlation coefficients (n = 8) among gross rates of soil-N cycling and microbial biomass for the top 0.05 m depth for the reference land uses across both soil landscapes in Jambi, Sumatra, Indonesia.

	NH_4_^+^ immobilization (mg N kg^-1^ day^-1^)	Gross nitrification (mg N kg^-1^ day^-1^)	NO_3_^-^ immobilization (mg N kg^-1^ day^-1^)	DNRA^1^ (mg N kg^-1^ day^-1^)	Microbial N (mg N kg^-1^)	Microbial C (mg C kg^-1^)	Microbial C:N
Lowland forest							
Gross N mineralization (mg N kg^-1^ day^-1^)	0.83*	-0.33	0.36	0.46	0.86*	0.81*	0.29
NH_4_^+^ immobilization (mg N kg^-1^ day^-1^)		-0.55	0.62	0.46	0.79*	0.74*	0.31
Gross nitrification (mg N kg^-1^ day^-1^)			-0.31	-0.20	-0.19	-0.21	-0.24
NO_3_^-^ immobilization (mg N kg^-1^ day^-1^)				0.57	0.55	0.64	0.45
DNRA^1^ (mg N kg^-1^ day^-1^)					0.28	0.30	-0.70
Microbial N (mg N kg^-1^)						0.98*	0.64^†^
Microbial C (mg C kg^-1^)							0.71*
Jungle rubber							
Gross N mineralization (mg N kg^-1^ day^-1^)	0.71*	0.00	0.74*	0.95*	0.76*	0.81*	0.41
NH_4_^+^ immobilization (mg N kg^-1^ day^-1^)		-0.05	0.98*	0.71*	0.81*	0.57	-0.22
Gross nitrification (mg N kg^-1^ day^-1^)			0.10	-0.07	0.26	-0.05	-0.32
NO_3_^-^ immobilization (mg N kg^-1^ day^-1^)				0.76*	0.90*	0.64^†^	-0.17
DNRA^1^ (mg N kg^-1^ day^-1^)					0.81*	0.88*	0.44
Microbial N (mg N kg^-1^)						0.83*	-0.01
Microbial C (mg C kg^-1^)							0.35

*P ≤ 0.05, and

^†^P ≤ 0.09

^1^Dissimilatory nitrate reduction to ammonium.

### Converted Land Uses: Oil Palm and Rubber Plantations

In both landscapes, soil pH was higher either in oil palm or rubber plantations compared to the reference land uses (P ≤ 0.05 to 0.09; Table 1). Soil organic C and total N stocks tended to be lower in the oil palm and rubber plantations than jungle rubber (i.e. in clay Acrisol soil) or than both reference land uses (i.e. loam Acrisol soil) (Table 1), although these trends were not statistically significant (P ≥ 0.10). The C:N ratios were lower in the converted land uses than the reference land uses in the loam Acrisol soil (P ≤ 0.05; Table 1). Base saturation and soil δ^15^N signatures were higher in the oil palm plantations than the reference land uses in the loam Acrisol soil (P ≤ 0.05 to 0.09; Table 1), and a similar trend was depicted in the clay Acrisol soil (Table 1) although not statistically significant (P ≥ 0.10). Extractable P was highest in the oil palm plantations, in the clay Acrisol (P ≤ 0.05; Table 1). Exchangeable Fe was lower in either oil palm or rubber plantations than the reference land uses in both landscapes (P ≤ 0.05; Table 1) and exchangeable Al showed a similar trend but was not statistically significant (P ≥ 0.10). Na was higher in the oil palm plantations than the reference land uses in the loam Acrisol soil (P ≤ 0.05; Table 1).

Gross N mineralization, NH_4_^+^ immobilization and NH_4_^+^ pools were lower in the converted land uses than the reference land uses in the clay Acrisol soil (P ≤ 0.05 to 0.09; Table 2), whereas these NH_4_^+^ transformation processes did not differ among land uses in the loam Acrisol soil (P ≥ 0.10). There were no differences in gross nitrification and NO_3_^-^ immobilization between converted and reference land uses in both landscapes (all P ≥ 0.10), but similar to that in the reference land uses the NO_3_^-^ transformation rates were smaller than the NH_4_^+^ transformation rates in the converted land uses (Table 2). It was also noticeable that in the rubber plantations, and to a lesser extent the jungle rubber, the NO_3_^-^ pools were lower than those in the forests and oil palm plantations in both landscapes (both P ≤ 0.05; Table 2). With regards to NO_3_^-^ retention processes, DNRA was less important (had lower rates) than NO_3_^-^ immobilization across all land uses in both landscapes (Table 2). However, its proportion to gross nitrification was large (55% to 71%) in the rubber plantations that had the lowest gross nitrification and NO_3_^-^ pools (Table 2). Microbial biomass C, N and C:N ratio did not differ (all P ≥ 0.10) between the converted and reference land uses in the loam Acrisol soil (Table 2), where microbial biomass was initially low (i.e. lower microbial C and N in the forest sites of loam than clay Acrisol soils). In the clay Acrisol soil, where the initial (or forests as reference land use) microbial biomass was large, microbial C and N were lower in the converted than reference land uses (P ≤ 0.05 to 0.09; Table 2).

In the rubber plantations, gross N mineralization was correlated positively with gross nitrification which, in turn, was correlated with NO_3_^-^ retention processes (immobilization and DNRA; Table 4). In the oil palm plantations, gross N mineralization also correlated positively with NH_4_^+^ immobilization and both as well as the NO_3_^-^ retention processes were directly correlated with either microbial C, N or both (Table 4).

**Table 4:** Spearman’s rank correlation coefficients (n = 8) among gross rates of soil-N cycling and microbial biomass for the top 0.05 m depth for the converted land uses across both soil landscapes in Jambi, Sumatra, Indonesia.

	NH_4_^+^ immobilization (mg N kg^-1^ day^-1^)	Gross nitrification (mg N kg^-1^ day^-1^)	NO_3_^-^ immobilization (mg N kg^-1^ day^-1^)	DNRA^1^ (mg N kg^-1^ day^-1^)	Microbial N (mg N kg^-1^)	Microbial C (mg C kg^-1^)	Microbial C:N
Rubber plantation							
Gross N mineralization (mg N kg^-1^ day^-1^)	0.38	0.71*	0.57	0.64	-0.24	-0.33	-0.10
NH_4_^+^ immobilization (mg N kg^-1^ day^-1^)		-0.17	0.50	-0.19	-0.19	0.21	0.52
Gross nitrification (mg N kg^-1^ day^-1^)			0.55	0.93*	0.17	-0.31	-0.60
NO_3_^-^ immobilization (mg N kg^-1^ day^-1^)				0.64^†^	0.10	0.05	-0.07
DNRA^1^ (mg N kg^-1^ day^-1^)					0.07	-0.21	-0.43
Microbial N (mg N kg^-1^)						0.62	-0.29
Microbial C (mg C kg^-1^)							0.50
Oil palm plantation							
Gross N mineralization (mg N kg^-1^ day^-1^)	0.76*	0.43	0.48	0.63^†^	0.67^†^	0.62	-0.74*
NH_4_^+^ immobilization (mg N kg^-1^ day^-1^)		0.55	0.88*	0.83*	0.86*	0.83*	-0.60
Gross nitrification (mg N kg^-1^ day^-1^)			0.62	0.17	0.52	0.38	-0.33
NO_3_^-^ immobilization (mg N kg^-1^ day^-1^)				0.71*	0.71*	0.67^†^	-0.36
DNRA^1^ (mg N kg^-1^ day^-1^)					0.63	0.71*	-0.42
Microbial N (mg N kg^-1^)						0.98*	-0.86*
Microbial C (mg C kg^-1^)							-0.83*

*P ≤ 0.05, and

^†^P ≤ 0.09

^1^Dissimilatory nitrate reduction to ammonium.

**Fig 3:**
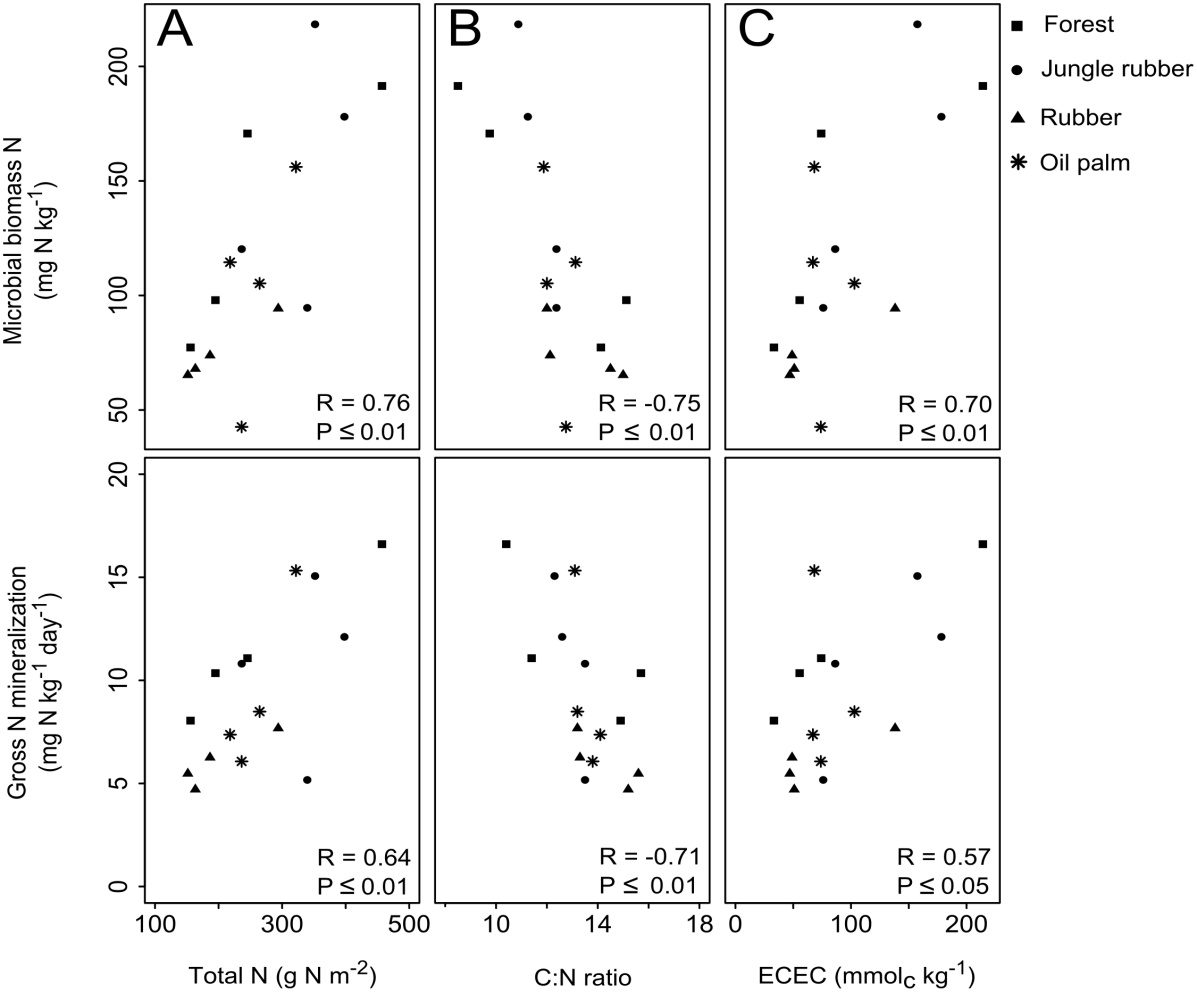
Relationships between microbial biomass N (top panels) and gross N mineralization (lower panels) with soil (A) total N, (B) C:N ratio and (C) effective cation exchange capacity (ECEC) across land-use types within the clay Acrisol soil (n = 16) in Jambi, Sumatra, Indonesia, assessed using Spearman’s rank correlations test.

We investigated whether the changes in soil biochemical characteristics due to land-use conversion affect changes in soil N availability. In the loam Acrisol soil that had lower soil fertility than the clay Acrisol soil (i.e. Table 1: lower pH, organic C, total N, pH, ECEC, base saturation or stocks of exchangeable bases and extractable P; see *Reference Land Uses* above), there were no correlations detected between soil biochemical characteristics and the index of soil N availability (i.e. gross N mineralization) and microbial biomass across land-use types. However, in the clay Acrisol soil, gross N mineralization and microbial biomass N were positively correlated with soil organic C (R = 0.51, P ≤ 0.05, n = 16 and R = 0.62, P ≤ 0.05, n = 16, respectively), total N and ECEC and negatively correlated with soil C:N ratio (Fig 3).

## Discussion

### Soil-N Cycling in the Reference Land Uses

The Acrisol soils in our reference land uses are characterized by low soil fertility (i.e. low ECEC and base saturation with conversely high Al saturation; Table 1) compared to other lowland forest soils on relatively less-weathered Cambisol and Nitisol soils [19]. Within this Acrisol soil group, clay content influences soil fertility, as indicated by the better soil biochemical characteristics in the clay than the loam Acrisol in the reference land uses (i.e. Table 1: pH, soil organic C, total N, extractable P, ECEC and base saturation or stocks of exchangeable bases). This corresponds with a study conducted across a textural gradient in lowland Amazonian forests on Ferralsol soils (which is a further weathered soil than our Acrisol soils) that found as clay content increases, C, N and ECEC also increases [26]. We found that the clay Acrisol soil had ample substrate for microbial communities, evidenced by its higher organic C and total N stocks that were mirrored with larger microbial biomass, NH_4_^+^ pool and NH_4_^+^ transformation rates than the loam Acrisol soil (Table 2).

Studies of gross soil-N cycling rates in lowland tropical forests and agroforestry systems are limited, and the majority of these studies were conducted in Latin American forests. In terms of making comparisons, we limit these to studies conducted in lowland tropical soils that utilized the ^15^N pool dilution technique with in-situ incubation and extraction of mineral N. NH_4_^+^ transformation rates in both reference land uses were comparable with those measured in a lowland forest in Costa Rica on Ferrasol soil (with gross N mineralization of 8 ± 1 mg N kg^-1^ day^-1^ [36]), but lower than those found in a lowland forest in Panama on more fertile Cambisol and Nitisol soils (with gross N mineralization of 29 ± 6 mg N kg^-1^ day^-1^ [19]). NO_3_^-^ transformation rates were comparable to those measured in Panamanian lowland forest soils (with gross nitrification of 0.8 ± 0.1 mg N kg^-1^ day^-1^ [19]).

The dominance of NH_4_^+^ pools and NH_4_^+^ transformation rates in our reference land uses over NO_3_^-^ pools and NO_3_^-^ transformation rates (Table 2) indicates a largely NH_4_^+^ based N economy, which is a common characteristic of the soil-N cycling in natural or unfertilized systems [13, 16, 19]. The main difference between our reference land uses, in terms of labile N pools, was the larger microbial C:N ratio and NO_3_^-^ pool in the forest than in the jungle rubber. A lower microbial C:N ratio typically indicates a bacterial dominated system [19], which may be a response to an increase in soil pH in the jungle rubber particularly in the clay Acrisol soil where microbial C:N ratio had also decreased (Table 2). Interestingly, the low NO_3_^-^ pool in jungle rubber, a feature that was much more distinct in the monoculture rubber plantations (see *Soil-N Cycling in the Converted Land Uses* below) is possibly due to the production of monoterpenes by rubber trees [37] that are known to reduce NO_3_^-^ levels and thus potentially alter soil N pathways [38].

The correlation between gross N mineralization and NH_4_^+^ immobilization in the reference land uses (Table 3) indicates tightly coupled NH_4_^+^ transformation processes. The correlations of NH_4_^+^ transformation rates with microbial C and N (Table 3) also suggest the influence of microbial biomass size on such efficient internal soil-N cycling. Such tightly coupled N production and retention processes were enhanced in the jungle rubber, as suggested by the additional correlations with NO_3_^-^ immobilization and DNRA (Table 3). This also supports the rapid conversion of NO_3_^-^ and hence possibly an even more efficient retention of N in this land-use type. In summary, the high gross N mineralization rates in these reference land uses, particularly in the clay Acrisol soil that had higher fertility relative to the loam Acrisol soil, are most likely linked to the high plant productivity (measured in the same reference land uses by Kotowska et al. [30]) and nutrient returns to the soil through decomposition, as well as the presence of N-fixing trees (e.g. S1 Table) that may have provided additional N to these systems [13, 21]. Thus, forests or agroforestry systems with minimal management practices like our jungle rubber, on highly-weathered soils that have high clay content maintained a relatively sustainable availability of N through an efficient cycling within and between the soil and vegetation.

### Soil-N Cycling in the Converted Land Uses

The changes in soil biochemical characteristics in the converted land uses, i.e. increases in pH (observed in both landscapes), base saturation, exchangeable Na, soil δ^15^N signatures and decrease in soil C:N ratio (all observed in loam Acrisol soil; Table 1), hinged on the legacy of biomass burning during conversion for both oil palm and rubber plantations and additionally by the influence of fertilization and liming for oil palm plantations. The effect of ashes (as source of inorganic nutrient ions) from biomass burning has been shown to remain decades after the initial burning [39] and our plantations were only 12–17 years old (S1 Table). A notable difference between the rubber and oil palm plantations was the extractable P (observed in the clay Acrisol soil; Table 1), which was highest in the fertilized oil palm plantations and lowest in the unfertilized rubber plantations. Al saturation in the clay Acrisol soil remained high in both rubber (73 ± 4%) and oil palm plantations (53 ± 7%; Table 1). Our Acrisol soil is still within the Al buffering range (through Al solubilization at pH 3–5 [40]), which may have resulted in a further decline in extractable P in the rubber plantations that had neither fertilization nor liming. Fertilization, liming and in part biomass burning may replenish nutrient stocks. However in unfertilized or non-agroforestry systems nutrient availability eventually declines within a decade of forest conversion as export from harvest and leaching exceeds the internal supply of nutrients in the soil [10–13]. We observed a trend of decreased (although statistically not detectable) soil organic C, total N and ECEC present in the oil palm or rubber plantations relative to either forest, jungle rubber or both (Table 1). Such decreases in soil biochemical characteristics corresponded with decreases in microbial C and N in the oil palm and rubber plantations (i.e. clay Acrisol soil; Table 1). These decreases in organic matter stocks in the converted land uses in the loam Acrisol soil were paralleled by a decrease in soil C:N ratio (Table 1). This suggests that the quality and quantity of organic matter input may have improved, making it more easily available for microbial use and vulnerable to losses. These losses are implied by the increased δ^15^N signatures in the soil of the oil palm plantations (Table 1), which reflects increased N losses (i.e. leaching of NH_4_^+^ and NO_3_^-^ and emissions of N-oxide gases increased following fertilization in oil palm sites; Kurniawan et al. unpublished data, Hassler et al. unpublished data) as isotopically light N is lost from the system leaving behind isotopically heavy soil N [41]. In summary, levels of exchangeable bases and extractable P were augmented in oil palm plantations mainly because of fertilization, whereas microbial biomass decreased in both converted land uses.

The decrease in microbial biomass in rubber and oil palm plantations in the clay Acrisol soil consequently affected soil-N cycling: gross N mineralization, NH_4_^+^ immobilization and NH_4_^+^ pools also decreased in these converted land uses (Table 2). Between the converted land uses in the clay Acrisol soil, where initial NH_4_^+^ pools and NH_4_^+^ transformation rates were large (i.e. reference forests; Table 2), these values were lowest in the unfertilized rubber plantations and intermediate in the fertilized oil palm plantations, thus supporting our second hypothesis. Fertilization in the oil palm plantations amended an otherwise eventual decrease in soil N availability in unfertilized systems [13]. Soil microbial biomass and NH_4_^+^ transformation rates in the converted land uses in the loam Acrisol soil were not as altered by land-use change as in the clay Acrisol soil, possibly because the microbial biomass and gross NH_4_^+^ transformation rates were initially low (i.e. reference forests; Table 2). We also found no correlations between the index of soil N availability with soil biochemical characteristics across land uses in the loam Acrisol soil, where soil fertility was lower than the clay Acrisol soil (Table 1). A similar pattern was observed from montane forest conversion in clay and sandy loam Cambisol soils in Sulawesi, Indonesia. The clay Cambisol soil that initially had large microbial biomass and gross N mineralization exhibits greater decreases upon conversion to unfertilized corn than the sandy loam Cambisol soil [13]. These findings illustrate that larger initial microbial biomass pools and soil-N cycling rates, tend to promote larger potential reductions upon land-use conversion.

There are no previous data reported on gross soil-N cycling rates for oil palm and rubber plantations. Hence, we can only compare our values with previous studies based on management practices (i.e. fertilized vs. unfertilized systems) and age of converted sites. Our unfertilized rubber plantations had three times higher NH_4_^+^ transformation rates than those found in unfertilized, 9-year continuously cultivated corn on clay Cambisol soil in Sulawesi, Indonesia [13]. NH_4_^+^ transformation rates in our fertilized oil palm plantations were similar to the fertilized, 1-year tree plantation on sandy loam Ferralsol soil in Costa Rica [36].

The rubber plantations, and to a lesser extent the jungle rubber, exhibited the lowest NO_3_^-^ pools (Table 2), which is probably due to allelochemicals produced by rubber trees called monoterpenes. Previous research has shown that fluxes of monoterpenes from monoculture rubber plantations can be ten times greater than natural forest and that alpha- and beta-pinene, two commonly produced monoterpenes by rubber trees, reduce nitrification in soils [37,38]. The production of monoterpenes by rubber trees is believed to provide a C source for soil microorganisms that increases their activity, and reduces NO_3_^-^ levels [38], possibly via immobilization and DNRA as suggested by the correlations between gross nitrification, NO_3_^-^ immobilization and DNRA (Table 4). This potentially conserves N in this land use where gross N mineralization was much reduced (i.e. clay Acrisol soil; Table 2).

The clay Acrisol soil that had initially high soil fertility (i.e. pH, soil organic C, total N, ECEC and base saturation or stocks of exchangeable bases in the reference land uses; Table 1), large microbial biomass and soil N availability (i.e. gross N mineralization in the reference land uses; Table 2) revealed that land-use change decreased soil fertility, subsequently decreasing microbial biomass and reduced soil N availability. This is evident by the correlations of gross N mineralization and microbial N with ECEC, total N and C:N ratio (Fig 3), which depicts the forest (except two sites) and jungle rubber within the upper range, the fertilized oil palm within the middle range and the unfertilized rubber within the lower range of soil N availability. Exceptions were found in two of the forest and one of the jungle rubber sites that were somewhat within the lower range. This was most likely due to their further distances from the other set of sites and thus the considerable spatial variation for these reference land uses in this soil landscape (Fig 1). Overall, the unfertilized rubber plantations exhibited the lowest fertility and microbial biomass (Fig 3; Table 2). The soil-N cycle was predominantly linked with microbial biomass (Tables 3 and 4), and without a robust microbial biomass pool, soil N availability in this land use decreased (i.e. lowest gross N mineralization; Table 2). The fertilized oil palm plantations showed slightly increased soil N availability along with increased soil biochemical characteristics (Fig 3; Table 2), mainly due to management practices. However, the low microbial biomass in the oil palm plantations (Table 2) suggests that soil fertility and N availability were perhaps only temporarily abated by fertilization and may not be as sustainable as in the original reference land uses. In these Acrisol soils, which generally have low fertility relative to other less-weathered tropical soils, management practices in converted oil palm and rubber plantations should aim at maintaining the levels of microbial biomass as found in the original land use, because the availability of N, and other nutrients, is dependent on its activity.

## Conclusions

In these highly weathered Acrisol soils, clay content affects the inherent fertility of the reference land uses, corroborating our first hypothesis. Between the converted land uses, soil N availability and microbial biomass decreased in the unfertilized rubber plantations, and were intermediary in the fertilized oil palm plantations, supporting our second hypothesis. Although fertilization has been shown to hinder the decline in soil N availability and other nutrients in our oil palm plantations, N fertilization was also associated with negative impacts on groundwater quality and soil-atmosphere greenhouse gas exchange (Kurniawan et al. unpublished data; Hassler et al. unpublished data), as evident from the increased soil δ^15^N signatures in the oil palm plantations. The typically low acid-buffering capacity of Acrisol soils makes them vulnerable to further decline in soil fertility with forest conversion to monoculture plantations, as evident by the decrease in extractable P in the unfertilized rubber plantations. Smallholders will likely become more dependent on fertilization and lime application in order to buffer the effects of additional acidity from N fertilization [19] and to enhance P availability in these Al saturated Acrisol soils that cover half of the land area of Sumatra. Overtime, N fertilization may lead to more negative impacts on soil fertility (i.e. further increases in Al solubility, base cation leaching losses and decreases in P solubility), surpassing fertilization’s impact on soil N availability. The availability of soil N and other nutrients in these oil palm plantations will rely heavily on fertilization and liming, which will incur additional costs to the smallholders, unless more sustainable management practices are employed. Further studies should test management trials on-site to screen for practices that will yield optimum benefits (e.g. harvest and profit) with maximum nutrient retention efficiency (or less nutrient losses) in the soil.

## Supporting Information

S1 Table Plantation age, tree species and vegetation characteristics. (PDF)

S2 Table Soil clay contents from 0.5 m down to 2-m depth. (PDF)
