# Supplementary material for: Soil Nitrogen-Cycling Responses to Conversion of Lowland Forests to Oil Palm and Rubber Plantations in Sumatra, Indonesia
Source: PLoS One. 2015 Jul 29;10(7):e0133325. doi: 10.1371/journal.pone.0133325 (PMC4519237; doi:10.1371/journal.pone.0133325)
Supplement: S1 Table — (PDF) [file pone.0133325.s001.pdf]

S1 Table. Plantation age and mean ± SE (n = 4) tree density, tree height, basal area, diameter at breast height (DBH) of trees ≥ 0.10 m DBH and the most common tree species for each land-use type within each soil landscape in Jambi, Sumatra, Indonesia.

	Land-use types			
Characteristic	Lowland rainforest	Jungle rubber	Rubber	Oil palm
	loam Acrisol soil			
Age range (years)	not determined (ND)	ND	14 - 17	12 - 16
Tree density (trees ha^{-1})[1]	658 ± 26	525 ± 60	440 ± 81	140 ±4
Tree height (m)[1]	20.0 ± 0.6	14.0 ± 0.2	13.4 ± 0.5	4.9 ± 0.6
Basal area (m^2 ha^{-1})[1]	30.7 ± 1.0	16.6 ± 0.4	12.2 ± 1.6	not applicable (NA)
DBH (cm)[1]	21.0 ± 0.5	16.8 ± 0.5	17.8 ± 1.2	NA
Most common tree species[2]	*Aporosa spp., Burseraceae spp., Dipterocarpaceae spp., Fabaceae spp., Gironniera spp., Myrtaceae spp., Plaquium spp., Porterandia sp., Shorea spp.*	*Alstonia spp., Artocarpus spp., Fabaceae sp., Hevea sp., Macaranga spp., Porterandia sp., Sloetia sp.*	*Hevea brasiliensis*	*Elaeis guineensis*
	clay Acrisol soil			
Age range (years)	ND	ND	7 - 16	9 - 13
Tree density (trees ha^{-1})[1]	471 ± 31	685 ± 72	497 ± 15	134 ± 6
Tree height (m)[1]	17.0 ± 0.5	15.2 ± 0.3	13.4 ± 0.1	4.0 ± 0.3
Basal area (m^2 ha^{-1})[1]	29.4 ± 1.7	21.1 ± 1.4	10.0 ± 1.4	NA
DBH (cm)[1]	23.0 ± 0.4	17.3 ± 0.6	15.2 ± 0.7	NA
Most common tree species[2]	*Archidendron sp., Baccaurea spp., Ochanostachys sp.*	*Artocarpus spp., Endospermum sp., Hevea sp., Macaranga spp.*	*Hevea brasiliensis*	*Elaeis guineensis*

[1] Kotowska et al. [30]

[2] Rembold et al. (unpublished data), based on trees found in five subplots (5 m x 5 m) of each replicate plot (50 m x 50 m) which had ≥ 20 individuals, except Fabaceae spp. which had ≤ 20 individuals.
