# Supplementary material for: Soil Nitrogen-Cycling Responses to Conversion of Lowland Forests to Oil Palm and Rubber Plantations in Sumatra, Indonesia
Source: PLoS One. 2015 Jul 29;10(7):e0133325. doi: 10.1371/journal.pone.0133325 (PMC4519237; doi:10.1371/journal.pone.0133325)
Supplement: S2 Table — (PDF) [file pone.0133325.s002.pdf]

S2 Table. Soil clay contents (means ± SE, n = 3) in various depth intervals for different land-use types within each soil landscape in Jambi, Sumatra, Indonesia.

	Land-use types			
Clay (%)	Lowland rainforest	Jungle rubber	Rubber plantation	Oil palm plantation
	loam Acrisol soil			
50 – 100 cm depth	28.7 ± 4.8	38.8 ± 9.0	45.1 ± 11.3	41.0 ± 3.1 B[1]
100 – 150 cm depth	33.3 ± 7.6	42.4 ± 9.9	46.1 ± 9.9	43.3 ± 2.8 B
150 – 200 cm depth	37.3 ± 8.6	44.5 ± 10.0	43.4 ± 6.5	47.6 ± 4.5 B
	clay Acrisol soil			
50 – 100 cm depth	34.9 ± 9.0 b†[2]	51.4 ± 12.6 ab†	36.8 ± 8.0 b†	69.7 ± 4.8 A b†
100 – 150 cm depth	39.0 ± 13.0	62.8 ± 12.6	40.8 ± 10.3	62.8 ± 3.6 A
150 – 200 cm depth	41.3 ± 11.2	46.6 ± 16.2	36.5 ± 10.8	63.3 ± 6.1 A

[1]Within column means followed by different capital letters indicate significant differences between soil landscapes within a land-use type (LME model with Fisher's LSD test at $P \leq 0.05$ and marginally significant at $^{\dagger}P \leq 0.09$).

[2]Within row means followed by different lower case letters indicate significant differences between land-use types within a soil landscape (LME model with Fisher's LSD test at $P \leq 0.05$ and marginally significant at $^{\dagger}P \leq 0.09$).
